# Systematic Review and Meta-Analysis of COVID-19 Vaccination Acceptance

**DOI:** 10.3389/fmed.2021.783982

**Published:** 2022-01-27

**Authors:** Mohd Noor Norhayati, Ruhana Che Yusof, Yacob Mohd Azman

**Affiliations:** ^1^Department of Family Medicine, School of Medical Sciences, Universiti Sains Malaysia, Kubang Kerian, Malaysia; ^2^Medical Practice Division, Ministry of Health, Federal Government Administrative Centre, Putrajaya, Malaysia

**Keywords:** COVID-19, vaccine, acceptance, meta-analysis, prevalence

## Abstract

**Introduction:**

Vaccination is an essential intervention to curb the coronavirus disease 2019 (COVID-19) pandemic. This review aimed to estimate the pooled proportion of COVID-19 vaccine acceptance worldwide.

**Methods:**

A systematic search of the MEDLINE (PubMed) database using “COVID-19,” “vaccine” and “acceptance” to obtain original research articles published between 2020 and July 2021. Only studies with full text and that were published in English were included. The Joanna Briggs Institute meta-analysis was used to assess the data quality. The meta-analysis was performed using generic inverse variance with a random-effects model using the Review Manager software.

**Results:**

A total of 172 studies across 50 countries worldwide were included. Subgroup analyses were performed with regard to vaccine acceptance, regions, population, gender, vaccine effectiveness, and survey time. The pooled proportion of COVID-19 vaccine acceptance was 61% (95% CI: 59, 64). It was higher in Southeast Asia, among healthcare workers, in males, for vaccines with 95% effectiveness, and during the first survey.

**Conclusion:**

COVID-19 vaccine acceptance needs to be increased to achieve herd immunity to protect the population from the disease. It is crucial to enhance public awareness of COVID-19 vaccination and improve access to vaccines.

**Systematic Review Registration:**

PROSPERO 2021, identifier CRD42021268645.

## Introduction

The coronavirus disease 2019 (COVID-19) pandemic is a global health topic of concern. As of August 2021, nearly 216 million COVID-19 cases have been reported globally, with the cumulative number of deaths being just under 4.5 million (1). The severe acute respiratory syndrome coronavirus 2 (SARS-CoV-2), which causes COVID-19, has changed over time. Change in the virus's properties may affect disease transmissibility, severity, and vaccine efficacy. The World Health Organization (WHO) has identified four SARS-CoV-2 variants of concern, namely alpha, beta, gamma, and delta, and five variants of interest, namely eta, iota, kappa, lambda, and mu (2).

In December 2020, the WHO approved the first vaccine, BNT162b2/COMIRNATY, which contains modified nucleoside mRNA that confers protection against COVID-19 (3). The United States Food and Drug Administration has granted emergency use authorization to use the COVID-19 vaccines and full approval for Pfizer vaccine to control the pandemic. The WHO has also recommended several vaccines for COVID-19, including COVISHIELD™, Janssen, Vaxzervria, Moderna, BIBP, CoronaVac, and AstraZeneca (3). The vaccines have the potential to create herd immunity without causing illness and complications (4). Herd immunity requires sufficient coverage and a large proportion of the population to be vaccinated.

However, the effectiveness of vaccination depends on the population's willingness to accept the vaccines. This urgent use of newly developed vaccines evokes a sense of vigilance in the general population. Many factors influence the population's acceptance of the vaccination, including risk perception of the disease, perception of vaccine safety and efficacy, public vaccination attitudes, past vaccination history, doctor's recommendations, costs, convenience, and sociodemographic characteristics (5).

Many studies have explored public perceptions of the COVID-19 vaccination program. The acceptance of COVID-19 vaccines varies among countries globally (6). Some studies showed a high level of vaccination acceptance (5, 7–12), while others reported an average level of acceptance (7, 8). An analysis of social media users indicated that of all participants involved, 36.4% in New York, 51.3% in London, 67.3% in São Paulo, 69.8% in Mumbai, and 76.8% in Beijing were willing to accept COVID-19 vaccines (9). An online survey in Arab countries reported that 83% of the study participants were hesitant to accept the vaccines because of their side effects, distrust in health care policies, the expedited production of vaccines, published studies, and vaccine-producing companies (10).

Determining the pooled estimated proportion of COVID-19 vaccination acceptance provides guidance to health authorities to prepare for an effective vaccination program. A successful and effective vaccination program can provide sufficient vaccination coverage in a population to achieve herd immunity and subsequently control the COVID-19 pandemic. Thus, this review aims to assess the estimated proportion of acceptance of the COVID-19 vaccines.

## Methods

### Study Design

A systematic review and meta-analysis of studies were conducted to assess the proportion of COVID-19 vaccination acceptance. The Preferred Reporting Items for Systematic Reviews and Meta-Analyses (PRISMA) guidelines (11) were followed to review articles of the studies. Ethics review and approval are not required for analyses of published data. This review was registered in PROSPERO 2021 (CRD42021268645).

### Eligibility Criteria

The criteria for inclusion include studies that report the proportion of COVID-19 vaccination acceptance. The acceptance of the vaccines included studies before or after the availability of COVID-19 vaccines. All types of COVID-19 vaccines were included in this review. Studies with cross-sectional, case-control, and cohort designs published in English from 2020 to 19 July 2021 were included. Case series/reports, conference papers, proceedings, articles available only in abstract form, editorial reviews, letters of communications, commentaries, systematic reviews, and qualitative studies were excluded. Articles in languages other than English were also be excluded.

### Information Sources

A systematic search was performed in the MEDLINE (PubMed) database for articles between 1 January 2020 and 19 July 2021.

### Search Strategy

The search was done using the generic free-text search terms “COVID-19” AND “vaccine” AND “acceptance.” All studies published from 2020 to 19 July 2021 were retrieved to assess their eligibility for inclusion in this study. The search was restricted to full-text only and English language articles. To find additional potentially eligible studies, reference lists of included citations were cross-checked.

### Selection Process

All records identified by our search strategy were exported to EndNote software. Duplicate articles were removed from the list. One independent reviewer screened the titles and abstracts of the identified articles. The full texts of eligible studies were obtained and read thoroughly to assess their suitability. The second reviewer validated the records. A third reviewer was consulted in the event of a conflict between the two reviewers. The search method was presented in the PRISMA flow chart showing the included studies and excluded with reasons for exclusion.

### Data Collection Process and Data Items

The data was extracted into Microsoft Excel (Microsoft Office Professional Plus 2016). The data included the first author, year of publication, study location, study design, setting, study population, sample size, proportion, and data to calculate effect estimates.

### Study Risk of Bias Assessment

Assessment of critical appraisal for data quality was assessed using the Joanna Briggs Institute (J.B.I.). Meta-Analysis for cross-sectional, case-control, and cohort studies (12). Two authors performed bias assessments independently.

### Effect Measures

The proportion of vaccines acceptance was reported in pooled estimate proportion with a 95% confidence interval. Terminology of vaccines acceptance refers to the willingness to be vaccinated, vaccines acceptability, desirability, demand, and positive attitudes toward the given vaccines.

### Synthesis Methods

The analysis was performed with Review Manager (RevMan) software (13). A generic inverse variance with a random-effects model was applied to pool the proportion of the studie's data. The heterogeneity was assessed by *I*^2^ statistic and used the guide as outlined: 0% to 40% might not be important; 30 to 60% may represent moderate heterogeneity; 50 to 90% may represent substantial heterogeneity; and 75 to 100% would be considerable heterogeneity (14). Subgroup analysis was performed based on WHO classification of world regions (African/American/Eastern Mediterranean/European/ South-East Asian/ Western Pacific), type of population (college students/ general adult/ healthcare/ high risk/ parents and caregivers), gender (male/ female), vaccines effectiveness (at 90% effective/ at 95% effective) and survey time (first survey/ second survey). The high-risk population represented people most at risk of exposure, such as teachers and school students, detained people, patients, and pregnant and breastfeeding women.

### Reporting Bias Assessment

The risk of bias was assessed by nine criteria (15): (1) appropriateness of sample frame (2) appropriateness of study participants sampled (3) adequate of sample size (4) description of study subjects and the setting (5) sample size justification, power description, or variance and effect estimates (6) valid methods for the identification of the condition (7) a standard and reliable condition measured (8) appropriateness of statistical analysis (9) adequate of response rate.

The criteria of the risk assessment were represented by “yes,” “no,” “unclear” or “not available.” The score for yes was one (1) and zero (0) for the rest. The risk of bias was considered low when the total score was more than 70%, moderate when 50–69%, and high when up to 0–49% (16).

## Results

### Study Selection

**Figure 1:**
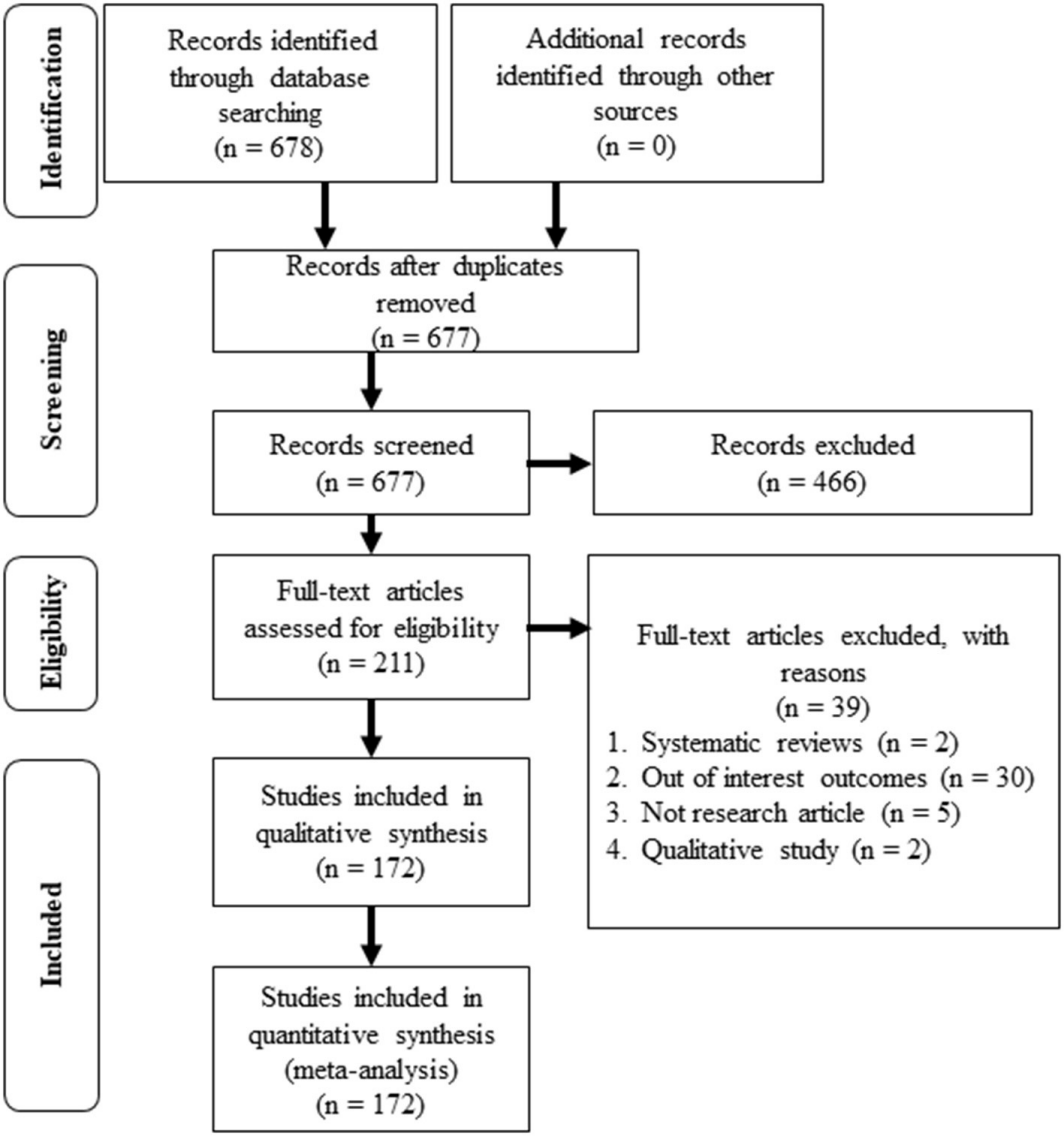
PRISMA flowchart of the review.

The primary search through the database had identified 678 studies. One duplicated study was removed, and 677 studies were screened for the titles and the abstracts. A total of 466 studies were excluded, and 211 full-text articles were assessed for eligibility. Thirty-nine studies were excluded for systematic review articles, out of interest outcomes, not research articles, and qualitative study articles. As a result, a total of 172 studies that met the inclusion and exclusion criteria were included for the review and meta-analysis (Figure 1).

### Study Characteristics

The articles of included studies were published in 2020 (*n* = 18) and in 2021 (*n* = 154). The studies involved 814,691 participants from 50 countries across six regions in the world represented as African region (*n* = 14) (17–30), American region (*n* = 34) (7, 31–63), Eastern Mediterranean region (*n* = 33) (8, 64–95), European region (*n* = 46) (96–141), South-East Asian region (*n* = 8) (5, 142–148) and Western Pacific region (*n* = 31) (149–179). There were two global studies (180, 181), one study combined countries in the European region and Western Pacific region (182), one study involved countries in the American region and Western Pacific region (183), one study involved the African region and Middle East countries (184) and one study involved countries in the African region, American region and South-East Asian region (185).

Most of the studies were designed as cross-sectional studies (*n* = 140), cohort studies (*n* = 4), descriptive studies (*n* = 23), longitudinal studies (*n* = 4) and combination of cross-sectional and longitudinal study (*n* = 1). A total of 28 studies applied probability sampling (random sampling, systematic sampling, stratified sampling, clustered sampling, and multistage sampling), 54 studies applied non-probability sampling (convenience sampling, quota sampling, purposive sampling, and snowball sampling), and 90 studies did not provide the sampling method (Supplementary Table 1).

Subgroup analysis involved the six regions in the world (African, American, Eastern Mediterranean, European, South-East Asian, and Western Pacific), population (college students, general adult population, healthcare workers, high-risk population, and parents and caregivers), gender, vaccines effectiveness (at 90 and 95%), and survey time.

### Risk of Bias in Studies

The J.B.I. quality assessment showed that 60 studies were at low risk of bias, 47 studies were at moderate bias, and 65 studies were at high risk of bias (Supplementary Table 2). All the studies were included. The pooled proportion of COVID-19 vaccines acceptance for the studies with low, moderate, and high risk of bias were 0.59 (95% CI: 0.54, 0.65), 0.61 (95% CI: 0.57, 0.66) and 0.63 (95% CI: 0.58, 0.68), respectively.

### Results of Total and Subgroup Studies

In Table 1, the total estimated proportion by COVID-19 vaccines acceptance pooled from 170 studies was 0.61 (95% CI: 0.59,0.64). The estimated pooled proportion by study designs of cohort study, cross sectional study, descriptive study, longitudinal study and combination study of longitudinal and cross-sectional were 0.77 (95% CI: 0.73, 0.81), 0.60 (95% CI: 0.57, 0.63), 0.69 (95% CI: 0.62, 0.75), 0.64 (95% CI: 0.49, 0.78) and 0.39 (95% CI: 0.37, 0.40) respectively. Meanwhile, the pooled proportion for probability sampling studies was 0.61 (95% CI: 0.55, 0.67) and 0.62 (95% CI: 0.57, 0.66) for non-probability sampling studies.

**Table 1:** The outcome measures of COVID-19 vaccines acceptance.

**Outcome**		**No. of studies**	**Proportion (95% CI)**	***I^**2**^* (%)**	***p*-value^a^**	**I^**2**^ diff (%)**	***p*-value^a^**
Vaccines acceptance		170	0.61 (0.59, 0.64)	100	*p* < 0.001		
**Subgroup:**							
Study designs	Total	170	0.61 (0.59, 0.64)	100	*p* < 0.001	99.1	p < 0.001
	Cohort	4	0.77 (0.73, 0.81)	90	*p* < 0.001		
	Cross sectional	138	0.60 (0.57, 0.63)	100	*p* < 0.001		
	Descriptive	23	0.69 (0.62, 0.75)	100	*p* < 0.001		
	Longitudinal	4	0.64 (0.49, 0.78)	99	*p* < 0.001		
	Combination	1	0.39 (0.37, 0.40)	NA	NA		
Sampling methods	Total	81	0.61 (0.58, 0.65)	100	*p* < 0.001	0	0.82
	Probability	27	0.61 (0.55, 0.67)	100	*p* < 0.001		
	Non-probability	54	0.62 (0.57, 0.66)	100	*p* < 0.001		
Regions	Total	165	0.62 (0.59, 0.64)	100	*p* < 0.001	71.5	0.004
	African	15	0.53 (0.39, 0.67)	100	*p* < 0.001		
	American	35	0.62 (0.57, 0.67)	100	*p* < 0.001		
	Eastern Mediterranean	33	0.52 (0.45, 0.59)	100	*p* < 0.001		
	European	46	0.65 (0.59, 0.71)	100	*p* < 0.001		
	South-East Asian	9	0.74 (0.64, 0.84)	100	*p* < 0.001		
	Western Pacific	32	0.66 (0.60, 0.73)	100	*p* < 0.001		
Population	Total	170	0.61 (0.59, 0.64)	100	*p* < 0.001	0	0.66
	College students	15	0.62 (0.52, 0.73)	100	*p* < 0.001		
	General adult	105	0.61 (0.58, 0.65)	100	*p* < 0.001		
	Healthcare workers	33	0.63 (0.56, 0.71)	100	*p* < 0.001		
	High risks	23	0.61 (0.52, 0.70)	100	*p* < 0.001		
	Parents and caregivers	7	0.52 (0.40, 0.65)	99	*p* < 0.001		
Gender	Total	89	0.60 (0.56, 0.65)	100	*p* < 0.001	44.8	0.18
	Male	89	0.64 (0.57, 0.71)	100	*p* < 0.001		
	Female	89	0.57 (0.48, 0.65)	100	*p* < 0.001		
Vaccines effectiveness	Total	6	0.71 (0.63, 0.79)	100	*p* < 0.001	36.5	0.21
	At 90% effective	3	0.62 (0.40, 0.84)	100	*p* < 0.001		
	At 95% effective	5	0.77 (0.69, 0.85)	100	*p* < 0.001		
Survey time	Total	7	0.65 (0.54, 0.75)	100	*p* < 0.001	0	0.64
	First survey	7	0.68 (0.56, 0.79)	99	*p* < 0.001		
	Second survey	7	0.62 (0.43, 0.81)	100	*p* < 0.001		

a *Test for overall effect*.

By subgroup analyses, the total proportion by regions was [0.62 (95% CI: 0.59, 0.64)] pooled from 165 studies, and the region with the highest proportion of COVID-19 vaccines acceptance was in South-East Asia [0.74 (95% CI: 0.64, 0.84)] which pooled from nine studies and the lowest vaccines acceptance was in Eastern Mediterranean [0.52 (95% CI: 0.45, 0.59)]. The total proportion by population was pooled from 170 studies [0.61 (95% CI: 0.59, 0.64)]. Population comparison showed that the proportion of COVID-19 vaccines acceptance was similar, which range 0.61 to 0.63 among college students, general adults, healthcare workers, and high-risk populations. However, the population of parents and caregivers was the lowest in the proportion of COVID-19 vaccines acceptance [0.52 (95% CI: 0.40, 0.65)].

The pooled proportion of COVID-19 vaccines acceptance by gender from 89 studies was [0.60 (95% CI: 0.56, 0.65)] and was higher in male [0.64 (95% CI: 0.57, 0.71)] compared to female [0.57 (95% CI: 0.48, 0.65)]. The 95% vaccines effectiveness showed higher in proportion of COVID-19 vaccines acceptance [0.77 (95% CI: 0.69, 0.85)] compared to 90% of vaccines effective [0.62 (95% CI: 0.40, 0.84)] with the total proportion by vaccines effectiveness was [0.71 (95% CI: 0.63, 0.79)] from six studies. The total pooled proportion from seven studies by survey time was 0.65 (95% CI: 0.54, 0.75). The proportion of COVID-19 vaccines acceptance was reduced in the second survey [0.62 (95% CI:0.43, 0.81)] compared to the first survey [0.68 (95% CI: 0.56, 0.79)]. In China and Australia, the surveys were repeated based on two epidemic phases, namely severe epidemic phase and well-contained phase (149, 171, 172). A study in Saudi Arabia performed the survey before and after the interim report of the efficacy rate of the RNA BNT162b2 vaccine (94).

All data for each outcome measure had considerable heterogeneity by the random-effects model (*I*^2^ > 99%). The heterogeneity of subgroups differences showed that the acceptance of the vaccine was with considerable heterogeneity; substantial heterogeneity for regions, moderate heterogeneity for gender and vaccines effectiveness. Heterogeneity might not be important for population and survey time (Table 1). The results were also presented in forest plots (Supplementary Figures 1–8).

## Discussion

Vaccination is an essential approach for tackling the COVID-19 pandemic by achieving herd immunity in the population. The effectiveness of this approach depends on vaccination acceptance in the population. In the current review, the pooled proportion of COVID-19 vaccine acceptance from 170 studies worldwide involving 50 countries was 61% (95% CI: 59, 64%). This finding was lower compared to a previous estimate of COVID-19 vaccine acceptance [73.31% (95% CI: 70.52%, 76.01%)], which involved 38 studies across 36 countries with limited data from low-income countries (186). Concerns about the vaccine's safety, efficacy, and side effects, trust in the government or related authorities (186), and religious beliefs (187) were primary factors that influenced vaccine acceptance. The subgroup analyses of this current review also determined variability in vaccine acceptance, which ranged from 52 to 77%.

The pooled proportion of COVID-19 vaccine acceptance among regions ranged from 52 to 74%, with Southeast Asia the highest and the Eastern Mediterranean the lowest. This result was supported by a review that reported vaccine acceptance of over 90% in Southeast Asia and the lowest proportions of acceptance in Middle East countries, with < 30% in Kuwait and Jordan (187). The low vaccine acceptance in the Middle East was related to widespread beliefs in conspiracies that negatively affected vaccination (188). Nevertheless, this current review also saw low vaccine acceptance in the African region. A study in Nigeria revealed that besides geographical location, which was associated with low vaccine acceptance, the other plausible reasons for this situation were low education levels, which led to poor health literacy, distrust in vaccines and the government, and cultural and religious beliefs, among others (18).

Vaccination acceptance was also higher in males than in females, which is in line with other reviews (187, 189). It was reported that males were less likely to believe conspiracy theories and more likely to perceive COVID-19 as dangerous (127). Females were found to express more concerns about the safety of vaccines and distrust in the quality and impartiality of vaccine information provided by healthcare professionals (190). However, a study with a higher proportion of females who accepted the COVID-19 vaccine reported that they perceived that vaccination was for the safety of families and communities. Widespread vaccination coverage could allow them to return to the previous work of routines and childcare arrangements (191).

The pooled proportion of COVID-19 vaccine acceptance for the population groups varied from 52 to 63%. Unsurprisingly, healthcare workers showed the highest proportion of vaccine acceptance. Since healthcare workers were among the first to receive COVID-19 vaccines, their attitude or perception toward COVID-19 vaccines would affect the other population's decisions to recommend the vaccination to friends, families, and their patients (192). Similar proportions (61 to 62%) were also seen in college students, the general adult public, and high-risk populations. As reported by other studies, challenges threatening vaccination uptake in a population include media misinformation, especially from social media (189), and widely broadcast rumors, myths, and inaccurate beliefs regarding vaccines by the anti-vaccine community (193). Confusing information may affect people's awareness of vaccination, especially those who lack sufficient knowledge concerning COVID-19 vaccines (194). For parents and caregivers, the proportion who accepted vaccination for their children was low (52%), and this might be influenced by insufficient clinical data on vaccine safety and efficacy on children (181) and their concern that young children are likelier to suffer side effects (195). Many COVID-19 vaccines are still not approved for children younger than 12, so parents may think vaccines are unsafe for young children.

The decision to accept COVID-19 vaccination was also influenced by vaccine effectiveness (196); this review showed that people were willing to take vaccines with higher efficacy. The interim result of a living systematic review showed that the effectiveness of the COVID-19 vaccine after one dose varied between 16.9 and 91.2%. While the effectiveness increased to between 61.7 and 98.6% after completing the second dose (197). However, insufficient evidence for COVID-19 vaccine effectiveness has been reported as a leading reason for reduced confidence in vaccines among the general population (37, 185).

The time during which the survey was conducted showed that the acceptance of the COVID-19 vaccine changed over time. All the studies showed evidence that the COVID-19 vaccine acceptance changed over time. The proportion of vaccine acceptance was reduced in the second survey. A global review of vaccine acceptance also showed a similar pattern. In a review, countries such as France, Italy, and China established a decreased proportion of vaccine acceptance in their second and third surveys.

Conversely, the United States showed an increased pattern of vaccine acceptance in the second and third surveys. The situation was different in the United Kingdom, which showed that the proportion was high in the first survey, increased further in the second survey, and then decreased for the third and fourth surveys before increasing again in the fifth survey. Still, in the fifth survey, the proportion of vaccine acceptance was not as high as in the first survey (187). It has also been reported that reduced vaccine acceptance is related to increased serious side effects of the vaccines (196).

Media and public service messaging, particularly fear appraisal-framed public service messages compared to safety benefits public service messages, influence willingness to obtain the COVID-19 vaccine. However, conspiracy theories circulated in the media by vaccination-averse people about vaccine side effects impact people's decision to get vaccinated (198). The hesitation in vaccination may be due to overestimating perceived risk in the COVID-19 pandemic, messaging fatigue, and desensitization caused by repeated exposure to information. Consequently, overloading information especially social media confuses people and impairs their ability to differentiate between reliable sources and incorrect information (189).

### Limitations of the Review

The search was limited to articles published in English only due to limited time, human, and financial resources to translate works published in other languages; this may have limited this review's generalizability. To have a more comprehensive assessment of the data, the authors decided to include all the available studies regardless of whether the quality of the data was low, moderate, or high, based on the assessment of the risk of bias. Furthermore, most of the research studies included in this review were cross-sectional studies, which can be thought of as visuals of vaccine hesitancy status in each country/region. They have different sampling strategies, which may explain some of the differences in vaccine acceptance rates reported in different studies from the same country. As a result, the findings should be regarded with caution, as they are unable to forecast future changes in vaccine acceptance rates. The other limitation was the sole dependence on the MEDLINE (PubMed) database in the search study. MEDLINE was reported as the best single source for retrieval of a systematic review, with an 89.7% inclusion rate that provided free-of-charge and open-access articles (199). However, it is also recommended to search extensively for studies using several databases to reduce possible biases in the included studies.

## Conclusion

The rate of COVID-19 vaccine acceptance varied by region, population type, gender, vaccine effectiveness, and survey time, with an overall pooled proportion of 61%. A high level of acceptance of vaccination is required to achieve herd immunity for the disease. Many vaccination campaigns and programs are available globally to enhance public awareness to access and accept the COVID-19 vaccine to reach herd immunity and control the pandemic.

## Data Availability Statement

The original contributions presented in the study are included in the article/Supplementary Materials, further inquiries can be directed to the corresponding author/s.

## Author Contributions

RCY and MNN: conceptualization, methodology, and validation. RCY: software, formal analysis, data curation, and writing—original draft preparation. MNN and RCY: writing—review and editing. MNN and YMA: visualization and supervision. All authors have read and agreed to the published version of the manuscript.

## Conflict of Interest

The authors declare that the research was conducted in the absence of any commercial or financial relationships that could be construed as a potential conflict of interest.

## Publisher's Note

All claims expressed in this article are solely those of the authors and do not necessarily represent those of their affiliated organizations, or those of the publisher, the editors and the reviewers. Any product that may be evaluated in this article, or claim that may be made by its manufacturer, is not guaranteed or endorsed by the publisher.

## Supplementary Material

Supplementary Figure 1 Forest plots of vaccines acceptance.

Supplementary Figure 2 Forest plots of vaccines acceptance by study designs.

Supplementary Figure 3 Forest plots of vaccines acceptance by sampling methods.

Supplementary Figure 4 Forest plots of vaccines acceptance by regions.

Supplementary Figure 5 Forest plots of vaccines acceptance by population.

Supplementary Figure 6 Forest plots of vaccines acceptance by gender.

Supplementary Figure 7 Forest plots of vaccines acceptance by vaccines effectiveness.

Supplementary Figure 8 Forest plots of vaccines acceptance by survey time.

Supplementary Table 1 Characteristics of the included studies.

Supplementary Table 2 J.B.I Critical appraisal checklist for studies reporting prevalence data.
