# Supplementary figures and images for: Systematic Review and Meta-Analysis of COVID-19 Vaccination Acceptance
Source: Front Med (Lausanne). 2022 Jan 27;8:783982. doi: 10.3389/fmed.2021.783982 (PMC8828741; doi:10.3389/fmed.2021.783982)

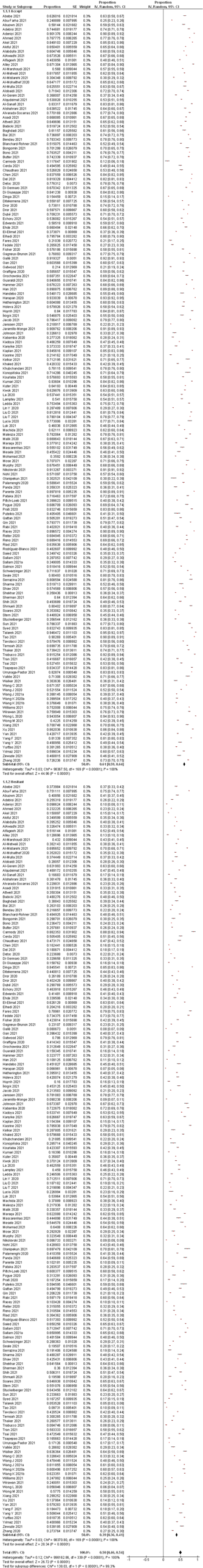

Supplement: Supplementary Figure 1 — Forest plots of vaccines acceptance. [file Image_1.TIF]

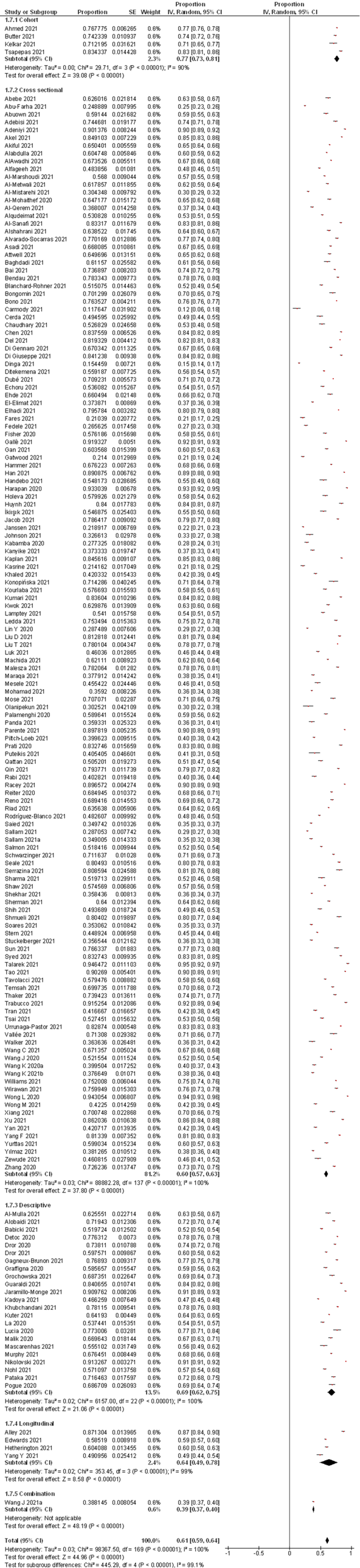

Supplement: Supplementary Figure 2 — Forest plots of vaccines acceptance by study designs. [file Image_2.TIF]

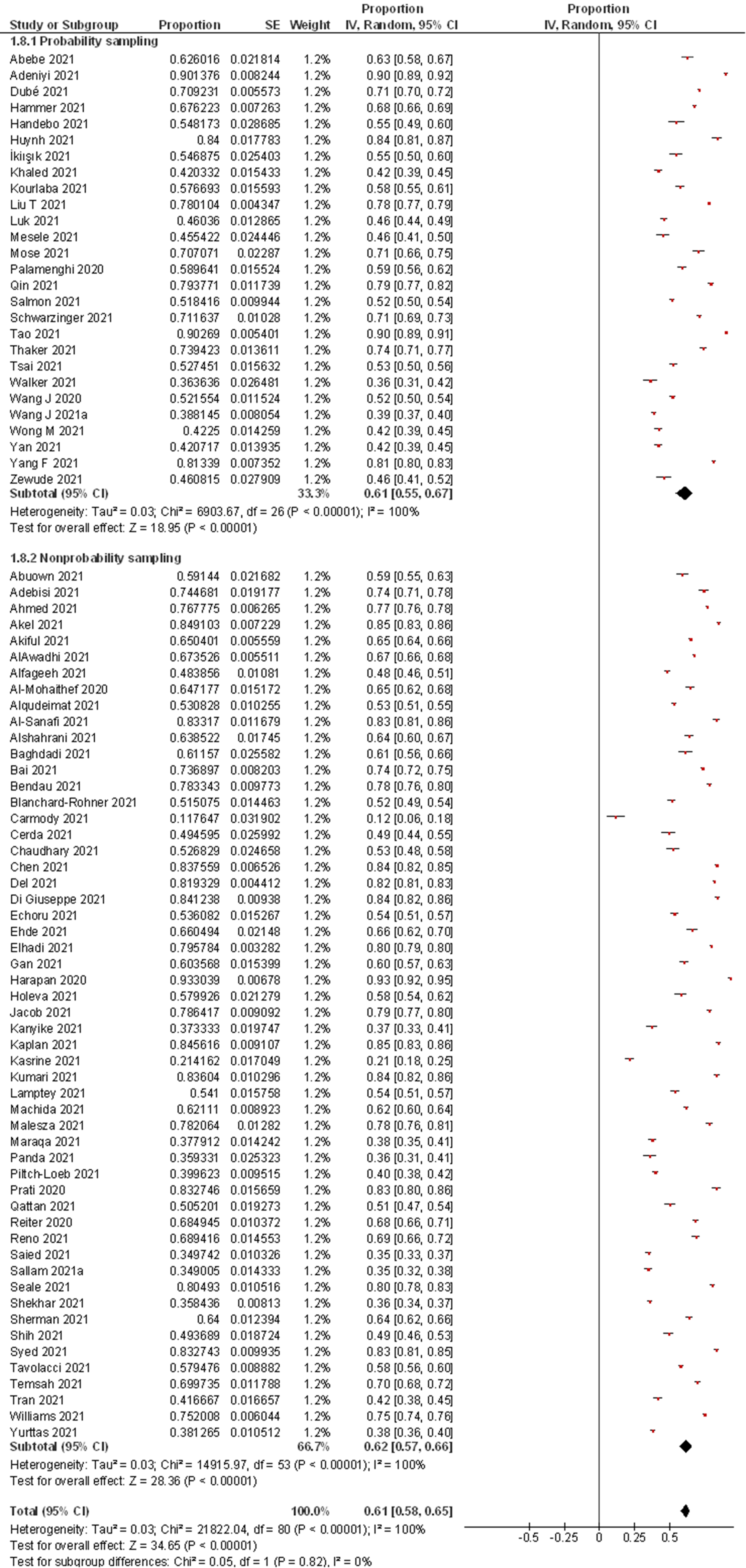

Supplement: Supplementary Figure 3 — Forest plots of vaccines acceptance by sampling methods. [file Image_3.TIF]

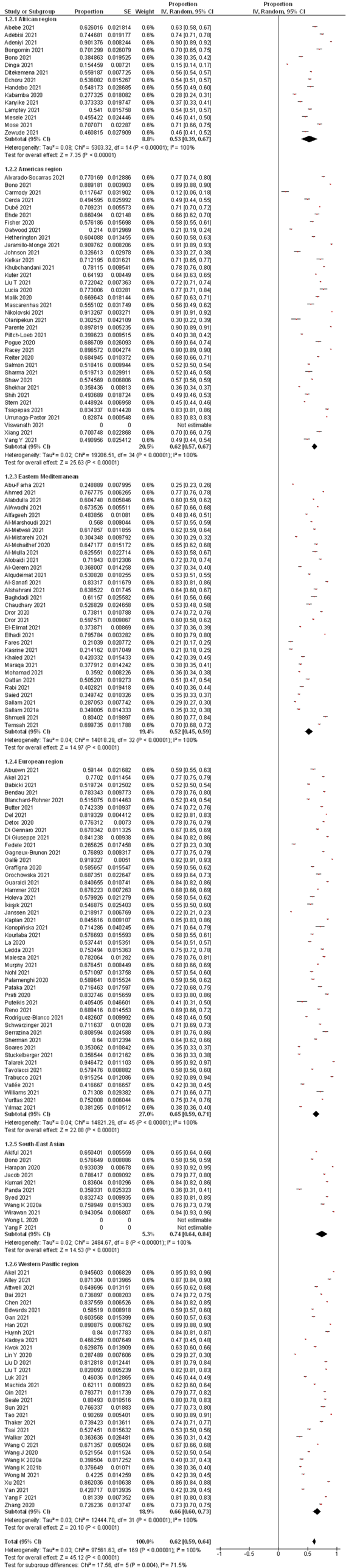

Supplement: Supplementary Figure 4 — Forest plots of vaccines acceptance by regions. [file Image_4.TIF]

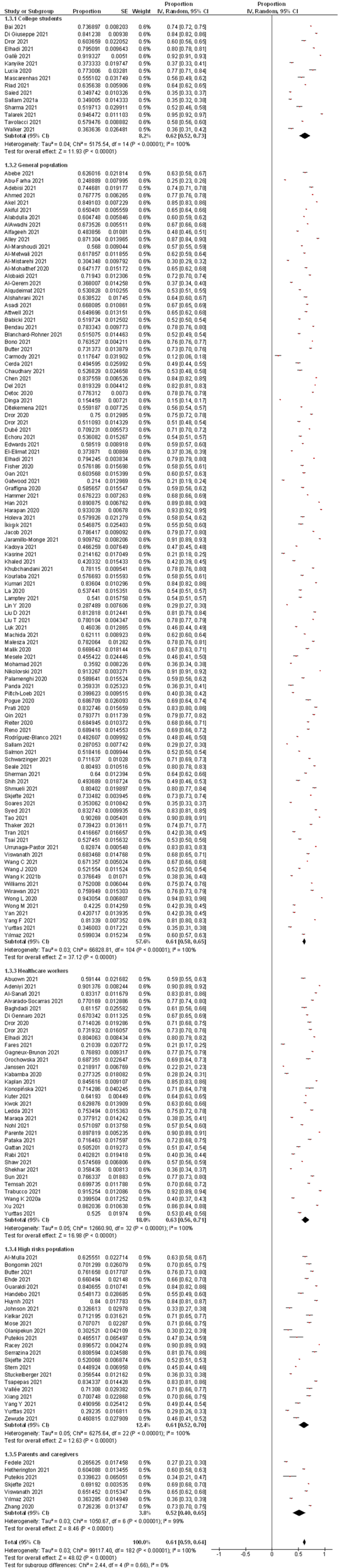

Supplement: Supplementary Figure 5 — Forest plots of vaccines acceptance by population. [file Image_5.TIF]

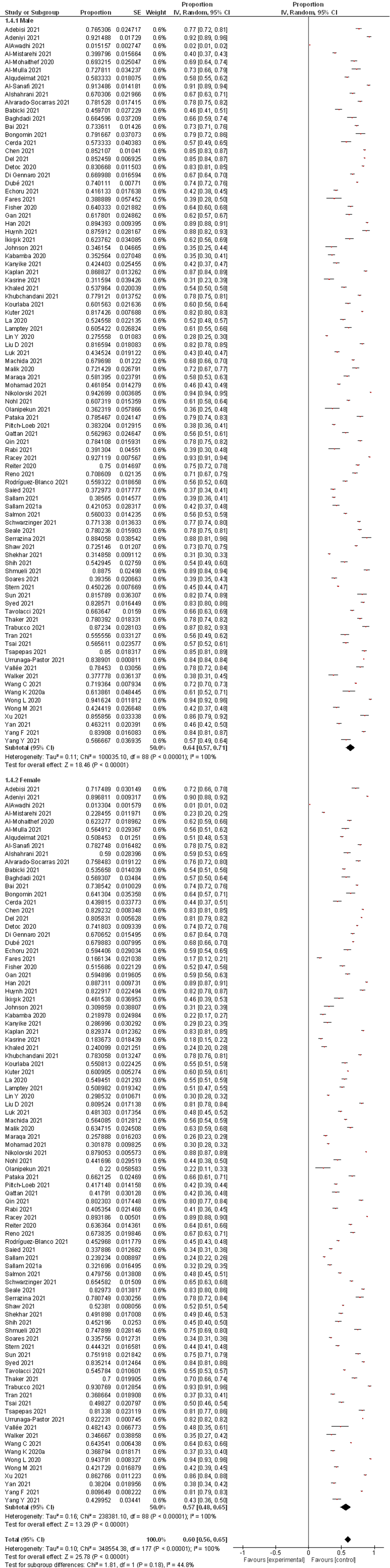

Supplement: Supplementary Figure 6 — Forest plots of vaccines acceptance by gender. [file Image_6.TIF]

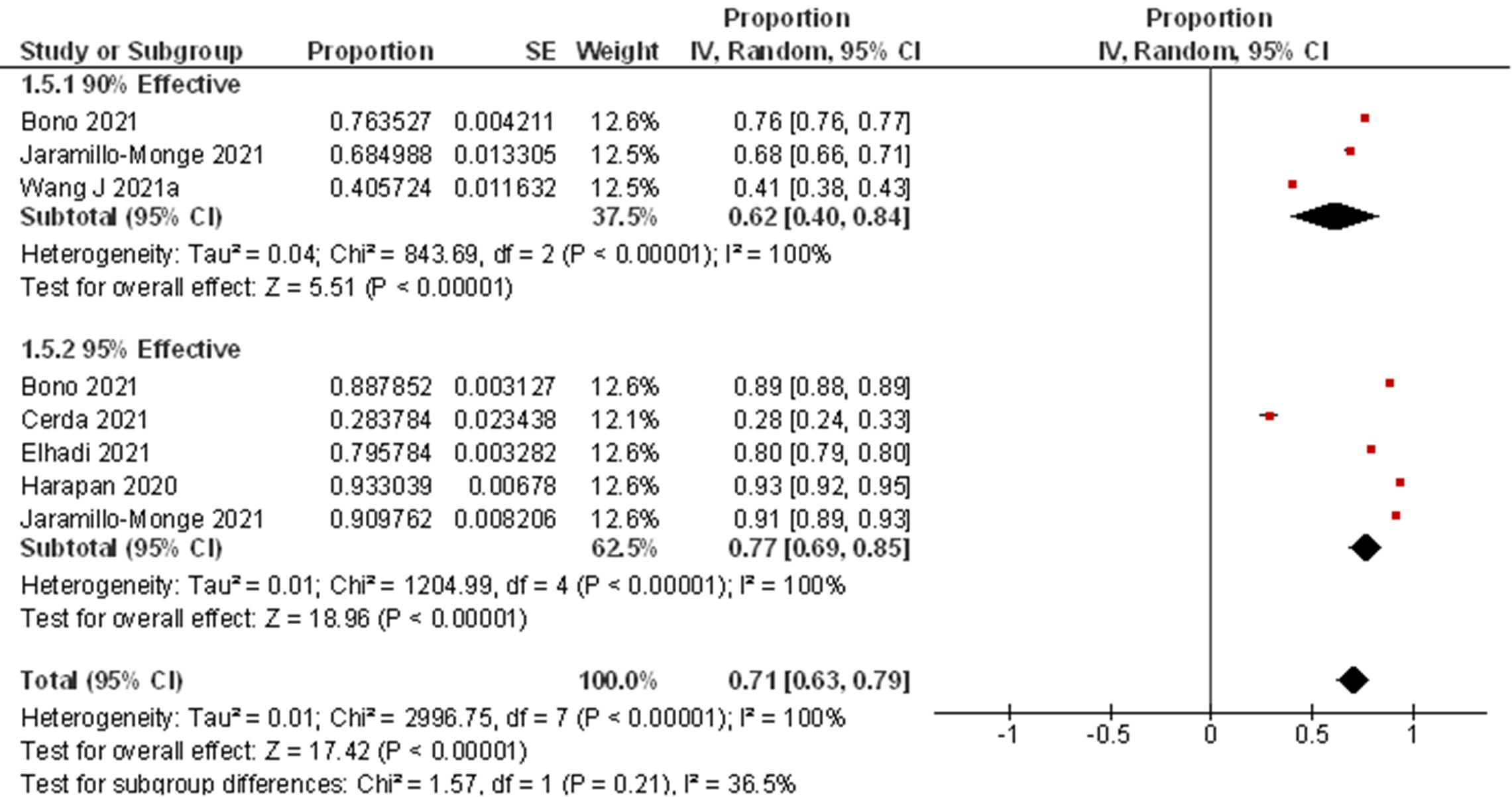

Supplement: Supplementary Figure 7 — Forest plots of vaccines acceptance by vaccines effectiveness. [file Image_7.TIF]

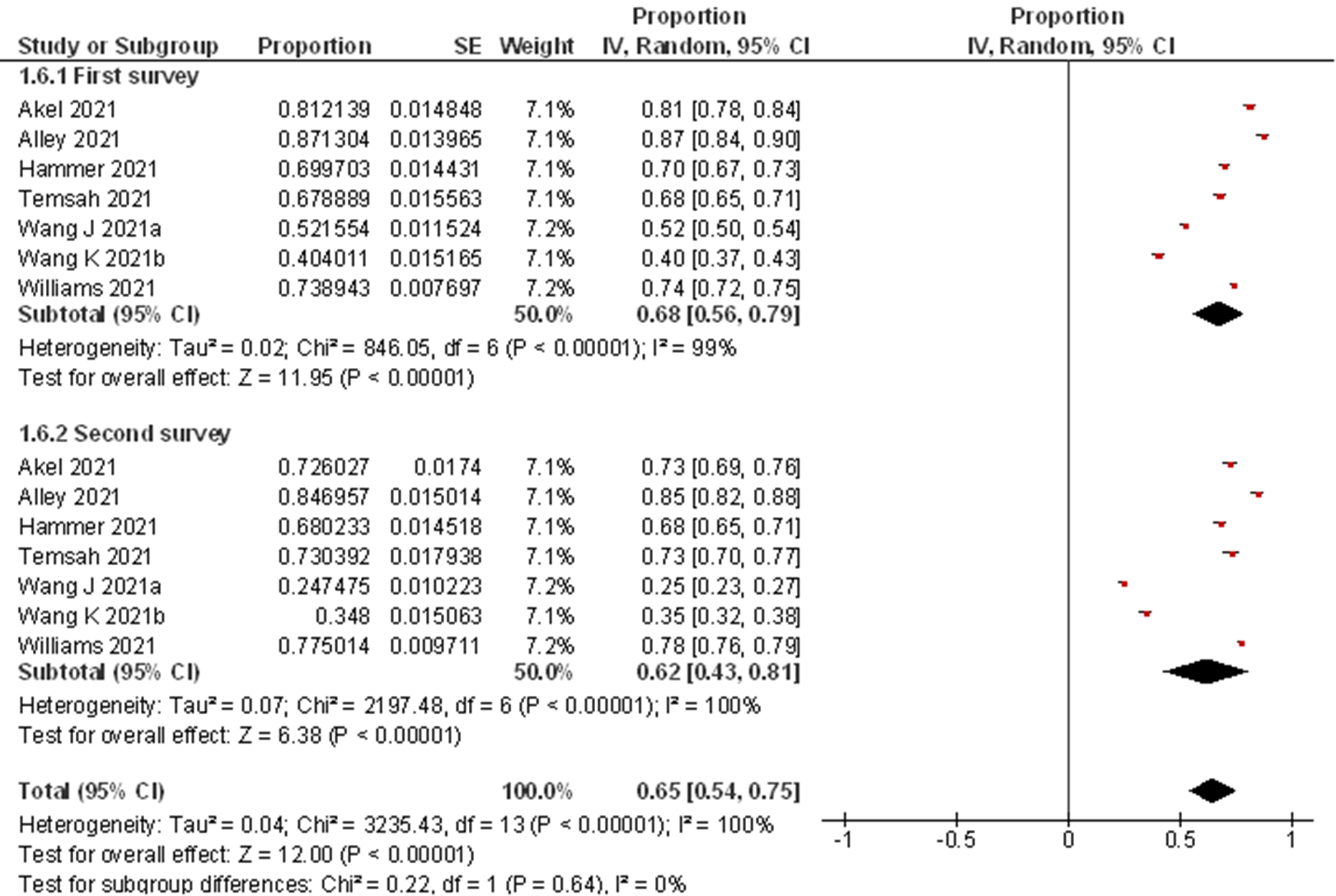

Supplement: Supplementary Figure 8 — Forest plots of vaccines acceptance by survey time. [file Image_8.TIF]
