# Supplementary material for: Systematic Review and Meta-Analysis of COVID-19 Vaccination Acceptance
Source: Front Med (Lausanne). 2022 Jan 27;8:783982. doi: 10.3389/fmed.2021.783982 (PMC8828741; doi:10.3389/fmed.2021.783982)
Supplement: Supplementary Table 1 — Characteristics of the included studies. [file Table_1.DOCX]

**Supplementary Table 1:** Characteristics of the included studies

| **Author** | **Year** | **Location** | **WHO_Region** | **Design** | **Sampling method** | **Population** | **N** |
| --- | --- | --- | --- | --- | --- | --- | --- |
| Abebe | 2021 | Ethopia | African | Cross-sectional | Multistage sampling | General population | 492 |
| Abu-Farha | 2021 | Middle Eastern Arab | Eastern Mediterranean | Cross-sectional | NA | General population | 2925 |
| Abuown | 2021 | UK | European | Cross-sectional | Convenience sampling | Healthcare workers | 514 |
| Adebisi | 2021 | Nigeria | African | Cross-sectional | Convenience sampling | General population | 517 |
| Adeniyi | 2021 | South Africa | African | Cross-sectional | Multi-stage cluster sampling. | Healthcare workers | 1308 |
| Ahmed | 2021 | Somalia | Eastern Mediterranean | Cohort study | Snowball sampling | General population | 4543 |
| Akel | 2021 | UK, China | European, Western Pacific | Cross-sectional | Quota sampling | General population | 2452 |
| Akiful Haque | 2021 | Bangladesh | South-East Asian | Cross-sectional | Convenience sampling | General population | 7,357 |
| Alabdulla | 2021 | Qatar | Eastern Mediterranean | Cross-sectional | NA | General population | 6993 |
| AlAwadhi | 2021 | Kuwait | Eastern Mediterranean | Cross-sectional | Convenience sampling | General population | 7241 |
| Alfageeh | 2021 | Saudi | Eastern Mediterranean | Cross-sectional | Simplified snowball sampling | General population | 2137 |
| Alley | 2021 | Australia | Western Pacific | Longitudinal study | NA | General population | 575 |
| Al-Marshoudi | 2021 | Oman | Eastern Mediterranean | Cross-sectional | NA | General population | 3000 |
| Al-Metwali | 2021 | Iraq | Eastern Mediterranean | Cross-sectional | NA | General population | 1680 |
| Al-Mistarehi | 2021 | Jordan | Eastern Mediterranean | Cross-sectional | NA | General population | 2,208 |
| Al-Mohaithef | 2020 | Saudi | Eastern Mediterranean | Cross-sectional | Snowball sampling | General population | 992 |
| Al-Mulla | 2021 | Qatar | Eastern Mediterranean | Descriptive study | NA | High risks | 454 |
| Alobaidi | 2021 | Saudi | Eastern Mediterranean | Descriptive study | NA | General population | 1333 |
| Al-Qerem | 2021 | Jordan | Eastern Mediterranean | Cross-sectional | NA | General population | 1144 |
| Alqudeimat | 2021 | Kuwait | Eastern Mediterranean | Cross-sectional | Snowball sampling | General population | 2,368 |
| Al-Sanafi | 2021 | Kuwait | Eastern Mediterranean | Cross-sectional | Convenience-based sampling | Healthcare workers | 1019 |
| Alshahrani, | 2021 | Saudi | Eastern Mediterranean | Cross-sectional | Snowball sampling | General population | 758 |
| Alvarado-Socarras | 2021 | Colombia | Americas | Cross-sectional | NA | Healthcare workers | 1066 |
| Asadi Faezi | 2021 | African and Middle East countries | African and Middle East countries | Cross-sectional | NA | General population | 1880 |
| Attwell | 2021 | Australia | Western Pacific | Cross-sectional | NA | General population | 1,316 |
| Babicki | 2021 | Poland | European | Descriptive study | NA | General population | 1597 |
| Baghdadi | 2021 | Saudi | Eastern Mediterranean | Cross-sectional | Convenience sampling | Healthcare workers | 363 |
| Bai | 2021 | China | Western Pacific | Cross-sectional | Snowball sampling | College students | 2,881 |
| Bendau | 2021 | Germany | European | Cross-sectional | Nonprobability sample | General population | 1777 |
| Blanchard-Rohner | 2021 | UK | European | Cross-sectional | Quota sampling | General population | 1194 |
| Bongomin | 2021 | Uganda | African | Cross-sectional | NA | High risks | 308 |
| Bono | 2021 | low & middle income countries (Brazil, Malaysia, Thailand, Bangladesh, Democratic Republic of Congo, Benin, Uganda, Malawi & Mali) | African, Americas, South-East Asian | Cross-sectional | NA | General population | 10,183 |
| Butter | 2021 | UK | European | Cohort study | NA | General population & high risks | 1599 |
| Carmody | 2021 | US | Americas | Cross-sectional | Convenience sampling | General population | 102 |
| Cerda | 2021 | Chile | Americas | Cross-sectional | Snowball sampling | General population | 370 |
| Chaudhary, | 2021 | Pakistan | Eastern Mediterranean | Cross-sectional | Convenience sampling | General population | 410 |
| Chen | 2021 | China | Western Pacific | Cross-sectional | Convenience sampling | General population | 3195 |
| Del Riccio | 2021 | Italy | European | Cross-sectional | Convenience sampling | General population | 7605 |
| Detoc | 2021 | France | European | Descriptive study | NA | General population | 3259 |
| Di Gennaro | 2021 | Italy | European | Cross-sectional | NA | Healthcare workers | 1723 |
| Di Giuseppe | 2021 | Italy | European | Cross-sectional | Convenience sampling | College students | 1518 |
| Dinga | 2021 | Cameroon | African | Cross-sectional | NA | General population | 2512 |
| Ditekemena | 2021 | Congo | African | Cross-sectional | NA | General population | 4131 |
| Dror | 2020 | Israel | Eastern Mediterranean | Descriptive study | NA | General population & healthcare workers | 1661 |
| Dror | 2021 | Israel | Eastern Mediterranean | Descriptive study | NA | General population, healthcare workers & college students | 2470 |
| Dubé | 2021 | Canada | Americas | Cross-sectional | Random sampling | General population | 6641 |
| Echoru | 2021 | Uganda | African | Cross-sectional | Snowball sampling | General population | 1067 |
| Edwards | 2021 | Australia | Western Pacific | Longitudinal study | NA | General population | 3,052 |
| Ehde | 2021 | US | Americas | Cross-sectional | Convenience sampling | High risks | 486 |
| El-Elimat | 2021 | Jordan | Eastern Mediterranean | Cross-sectional | NA | General population | 3100 |
| Elhadi | 2021 | Libya | Eastern Mediterranean | Cross-sectional | Snowball sampling | General population, healthcare workers & college students | 15,087 |
| Fares | 2021 | Egypt | Eastern Mediterranean | Cross-sectional | NA | Healthcare workers | 385 |
| Fedele | 2021 | Italy | European | Cross-sectional | NA | Parents & caregivers | 640 |
| Fisher | 2020 | US | Americas | Cross-sectional | NA | General population | 991 |
| Gagneux-Brunon | 2021 | France | European | Descriptive study | NA | Healthcare workers | 2047 |
| Gallè | 2021 | Italy | European | Cross-sectional | NA | College students | 2851 |
| Gan | 2021 | china | Western Pacific | Cross-sectional | Convenience sampling | General population | 1009 |
| Gatwood | 2021 | US | Americas | Cross-sectional | NA | General population | 1000 |
| Graﬃgna | 2020 | Italy | European | Descriptive study | NA | General population | 1004 |
| Grochowska | 2021 | Poland | European | Descriptive study | NA | Healthcare workers | 419 |
| Guaraldi | 2021 | Italy | European | Descriptive study | NA | High risks | 1161 |
| Hammer | 2021 | Finland | European | Cross-sectional | Random sampling | General population | 4151 |
| Han | 2021 | China | Western Pacific | Cross-sectional | NA | General population | 2126 |
| Handebo | 2021 | Ethiopia | African | Cross-sectional | Stratified simple random sampling | High risks | 301 |
| Harapan | 2020 | Indonesia | South-East Asian | Cross-sectional | Simplified-snowball sampling | General population | 1,359 |
| Hetherington | 2021 | Canada | Americas | Longitudinal study | NA | Parents & caregivers | 1321 |
| Holeva | 2021 | Greece | European | Cross-sectional | Convenience sampling | General population | 538 |
| Huynh | 2021 | Vietnam | Western Pacific | Cross-sectional | Systematic random sampling | High risks | 425 |
| İkiışık | 2021 | Turkey | European | Cross-sectional | Simple random sampling | General population | 384 |
| Jacob | 2021 | India | South-East Asian | Cross-sectional | Snowball sampling | General population | 2032 |
| Janssen | 2021 | France | European | Cross-sectional | NA | Healthcare workers | 3732 |
| Jaramillo-Monge | 2021 | Ecuador | Americas | Descriptive study | NA | General population | 1219 |
| Johnson | 2021 | US | Americas | Cross-sectional | NA | High risks | 248 |
| Kabamba | 2020 | Congo | African | Cross-sectional | NA | Healthcare workers | 613 |
| Kadoya | 2021 | Japan | Western Pacific | Descriptive study | NA | General population | 4253 |
| Kanyike | 2021 | Uganda | African | Cross-sectional | Convenience sampling | College students | 600 |
| Kaplan | 2021 | Turkey | European | Cross-sectional | Snowball sampling | Healthcare workers | 1574 |
| Kasrine | 2021 | Lebanon | Eastern Mediterranean | Cross-sectional | Snowball sampling | General population | 579 |
| Kelkar | 2021 | US | Americas | Cohort study | NA | High risks | 205 |
| Khaled | 2021 | Qatar | Eastern Mediterranean | Cross-sectional | Probability sampling | General population | 1023 |
| Khubchandani | 2021 | US | Americas | Descriptive study | NA | General population | 1878 |
| Konopi´nska | 2021 | Poland | European | Cross-sectional | NA | Healthcare workers | 126 |
| Kourlaba | 2021 | Greece | European | Cross-sectional | Systematic sampling | General population | 1004 |
| Kumari | 2021 | India | South-East Asian | Cross-sectional | Snowball sampling | General population | 1293 |
| Kuter | 2021 | US | Americas | Descriptive study | NA | Healthcare workers | 11,400 |
| Kwok | 2021 | Hong Kong | Western Pacific | Cross-sectional | NA | Healthcare workers | 1205 |
| La Vecchia | 2020 | Italy | European | Descriptive study | NA | General population | 1055 |
| Lamptey | 2021 | Ghana | African | Cross-sectional | Snowball sampling | General population | 1000 |
| Ledda | 2021 | Italy | European | Cross-sectional | NA | Healthcare workers | 787 |
| Lin | 2020 | China | Western Pacific | Cross-sectional | NA | General population | 3,541 |
| Liu D | 2021 | China | Western Pacific | Cross-sectional | NA | General population | 983 |
| Liu T | 2021 | China, US | Americas, Western Pacific | Cross-sectional | Stratified sampling | General population | 9077 |
| Lucia | 2020 | US | Americas | Descriptive study | NA | College students | 163 |
| Luk | 2021 | Hong Kong | Western Pacific | Cross-sectional | Random sampling | General population | 1501 |
| Machida | 2021 | Japan | Western Pacific | Cross-sectional | Quota sampling | General population | 2956 |
| Malesza | 2021 | Germany | European | Cross-sectional | Convenience sampling | General population | 1037 |
| Malik | 2020 | US | Americas | Descriptive study | NA | General population | 672 |
| Maraqa | 2021 | Palestine | Eastern Mediterranean | Cross-sectional | Convenience sampling | Healthcare workers | 1159 |
| Mascarenhas | 2021 | US | Americas | Descriptive study | NA | College students | 245 |
| Mesele | 2021 | Ethiopia | African | Cross-sectional | Random sampling | General population | 415 |
| Mohamad | 2021 | Syria | Eastern Mediterranean | Cross-sectional | NA | General population | 3402 |
| Mose | 2021 | Ethiopia | African | Cross-sectional | Systematic random sampling | High risks | 396 |
| Murphy | 2021 | Ireland, UK | European | Descriptive study | NA | General population | 3066 |
| Nikolovski | 2021 | US | Americas | Descriptive study | NA | General population | 7,402 |
| Nohl | 2021 | Germany | European | Descriptive study | NA | Healthcare workers | 1294 |
| Olanipekun | 2021 | US | Americas | Cross-sectional | NA | High risks | 119 |
| Palamenghi | 2020 | Italy | European | Cross-sectional | Stratified random sampling | General population | 1004 |
| Panda | 2021 | India | South-East Asian | Cross-sectional | Snowball sampling | General population | 359 |
| Parente | 2021 | US | Americas | Cross-sectional | NA | Healthcare workers | 3347 |
| Pataka | 2021 | Greece | European | Descriptive study | NA | Healthcare workers | 656 |
| Piltch-Loeb | 2021 | US | Americas | Cross-sectional | Purposive sampling | General population | 2,650 |
| Pogue | 2020 | US | Americas | Descriptive study | NA | General population | 316 |
| Prati | 2020 | Italy | European | Cross-sectional | Snowball sampling | General population | 568 |
| Puteikis | 2021 | Lithuania | European | Cross-sectional | NA | High risks & parents & caregivers | 111 |
| Qattan | 2021 | Saudi | Eastern Mediterranean | Cross-sectional | Simplified-snowball sampling | Healthcare workers | 673 |
| Qin | 2021 | China | Western Pacific | Cross-sectional | Random sampling | General population | 1188 |
| Rabi | 2021 | Palestine | Eastern Mediterranean | Cross-sectional | NA | Healthcare workers | 638 |
| Racey | 2021 | Canada | Americas | Cross-sectional | NA | High risks | 5076 |
| Reiter | 2020 | US | Americas | Cross-sectional | Convenience sampling | General population | 2006 |
| Reno | 2021 | Italy | European | Cross-sectional | Quota-based sampling | General population | 1011 |
| Riad | 2021 | Global | Global | Cross-sectional | NA | College students | 6639 |
| Rodríguez-Blanco | 2021 | Spain | European | Cross-sectional | NA | General population | 2501 |
| Saied | 2021 | Egypt | Eastern Mediterranean | Cross-sectional | Convenience sampling | College students | 2133 |
| Sallam | 2021 | Arab countries | Eastern Mediterranean | Cross-sectional | NA | General population | 3414 |
| Sallam_b | 2021 | Jordan | Eastern Mediterranean | Cross-sectional | Chain-referral sampling | College students | 1106 |
| Salmon | 2021 | US | Americas | Cross-sectional | Probability quota sampling | General population | 2525 |
| Schwarzinger | 2021 | France | European | Cross-sectional | Stratified random sampling | General population | 1942 |
| Seale | 2021 | Australia | Western Pacific | Cross-sectional | Quota sampling | General population | 1420 |
| Serrazina | 2021 | Portugal | European | Cross-sectional | NA | High risks | 256 |
| Sharma | 2021 | US | Americas | Cross-sectional | NA | College students | 279 |
| Shaw | 2021 | US | Americas | Cross-sectional | NA | Healthcare workers | 5277 |
| Shekhar | 2021 | US | Americas | Cross-sectional | Snowball sampling | Healthcare workers | 3479 |
| Sherman | 2021 | UK | European | Cross-sectional | Quota sampling | General population | 1500 |
| Shih | 2021 | US | Americas | Cross-sectional | Quota sampling | General population | 713 |
| Shmueli | 2021 | Israel | Eastern Mediterranean | Cross-sectional | NA | General population | 398 |
| Skjefte | 2021 | Global | Global | Cross-sectional | NA | General population, high risks & parents & caregivers | 17,871 |
| Soares | 2021 | Portugal | European | Cross-sectional | NA | General population | 1943 |
| Stern | 2021 | US | Americas | Cross-sectional | NA | High risks | 5,110 |
| Stuckelberger | 2021 | Switzerland | European | Cross-sectional | NA | High risks | 1551 |
| Sun | 2021 | China | Western Pacific | Cross-sectional | NA | Healthcare workers | 505 |
| Syed Alwi | 2021 | Malaysia | South-East Asian | Cross-sectional | Snowball sampling | General population | 1411 |
| Talarek | 2021 | Poland | European | Cross-sectional | NA | College students | 411 |
| Tao | 2021 | China | Western Pacific | Cross-sectional | Stratified random sampling | General population | 3011 |
| Tavolacci | 2021 | France | European | Cross-sectional | Convenience sampling | College students | 3089 |
| Temsah | 2021 | Saudi | Eastern Mediterranean | Cross-sectional | Convenience sampling | Healthcare workers | 1512 |
| Thaker | 2021 | New Zealand | Western Pacific | Cross-sectional | Stratified random sampling | General population | 1040 |
| Trabucco Aurilio | 2021 | Italy | European | Cross-sectional | NA | Healthcare workers | 531 |
| Tran | 2021 | Russia | European | Cross-sectional | Snowball sampling | General population | 876 |
| Tsai | 2021 | Taiwan | Western Pacific | Cross-sectional | Stratified sampling | General population | 1020 |
| Tsapepas | 2021 | US | Americas | Cohort study | NA | High risks | 664 |
| Urrunaga-Pastor | 2021 | Latin America | Americas | Cross-sectional | NA | General population | 472,521 |
| Vallée | 2021 | France | European | Cross-sectional | NA | High risks | 237 |
| Viswanath | 2021 | US | Americas | Cross-sectional | Random sampling | General population & parents & caregivers | 992 |
| Walker | 2021 | China | Western Pacific | Cross-sectional | Cluster random sampling | College students | 330 |
| Wang C | 2021 | China | Western Pacific | Cross-sectional | NA | General population | 8742 |
| Wang J_a | 2020 | China | Western Pacific | Cross-sectional | Stratified random sampling | General population | 1879 |
| Wang J_b | 2021 | China | Western Pacific | Longitudunal & cross-sectional study | Stratified random sampling | General population | 3661 |
| Wang K_a | 2020 | Hong Kong | Western Pacific | Cross-sectional | NA | Healthcare workers | 806 |
| Wang K_b | 2021 | Hong Kong | Western Pacific | Cross-sectional | NA | General population | 1196 |
| Williams | 2021 | Scotland | European | Cross-sectional | Convenience sampling | General population | 5105 |
| Wirawan | 2021 | Indonesia | South-East Asian | Cross-sectional | NA | General population | 779 |
| Wong L | 2020 | Malaysia | South-East Asian | Cross-sectional | NA | General population | 1159 |
| Wong M | 2021 | Hong Kong | Western Pacific | Cross-sectional | Random sampling | General population | 1200 |
| Xiang | 2021 | US | Americas | Cross-sectional | NA | High risks | 401 |
| Xu | 2021 | China | Western Pacific | Cross-sectional | NA | Healthcare workers | 1051 |
| Yan | 2021 | Hong Kong | Western Pacific | Cross-sectional | Random sampling | General population | 1255 |
| Yang F | 2021 | China | Western Pacific | Cross-sectional | Multistage stratified sampling | General population | 2808 |
| Yang Y | 2021 | US | Americas | Longitudunal study | NA | High risks | 387 |
| Yılmaz | 2021 | Turkey | European | Cross-sectional | NA | General population & parents & caregivers | 1035 |
| Yurttas | 2021 | Turkey | European | Cross-sectional | Snowball sampling | General population, healthcare workers & high risks | 2135 |
| Zewude | 2021 | Ethiopia | African | Cross-sectional | Probability proportion | High risks | 319 |
| Zhang | 2020 | China | Western Pacific | Cross-sectional | NA | Parents & caregivers | 1052 |

N = total number; NA = Not Available
