# Supplementary material for: Systematic Review and Meta-Analysis of COVID-19 Vaccination Acceptance
Source: Front Med (Lausanne). 2022 Jan 27;8:783982. doi: 10.3389/fmed.2021.783982 (PMC8828741; doi:10.3389/fmed.2021.783982)
Supplement: Supplementary Table 2 — J.B.I Critical appraisal checklist for studies reporting prevalence data. [file Table_2.DOCX]

Table 2: J.B.I Critical Appraisal Checklist for Studies Reporting Prevalence Data

| **Criteria (Yes/No/Unclear/NA)**  **Yes = 1**  **No/Unclear/NA = 0** | Was the sample frame appropriate to address the target population? | Were study participants sampled in an appropriate way? | Was the sample size adequate? | Were the study subjects and the setting described in detail? | Was a sample size justification, power description, or variance and effect estimates provided? | Were valid methods used for the identification of the condition? | Was the condition measured in a standard, reliable way for all participants? | Was there appropriate statistical analysis? | Was the response rate adequate, and if not, was the low response rate managed appropriately? | **Total quality score**  **n (%)** | Risk of Bias |
| --- | --- | --- | --- | --- | --- | --- | --- | --- | --- | --- | --- |
| Abebe | 1 | 1 | 1 | 1 | 1 | 1 | 1 | 1 | 1 | 9(100) | low |
| Abu-Farha | 0 | 0 | 0 | 1 | 0 | 1 | 1 | 1 | 0 | 4(44.4) | high |
| Abuown | 0 | 1 | 0 | 1 | 0 | 1 | 1 | 1 | 0 | 5(55.6) | moderate |
| Adebisi | 0 | 1 | 1 | 1 | 1 | 1 | 1 | 1 | 1 | 8(88.9) | low |
| Adeniyi | 0 | 1 | 0 | 1 | 0 | 1 | 1 | 1 | 0 | 5(55.6) | moderate |
| Ahmed | 0 | 1 | 0 | 1 | 0 | 1 | 1 | 1 | 0 | 5(55.6) | moderate |
| Akel | 0 | 1 | 0 | 1 | 0 | 1 | 1 | 1 | 0 | 5(55.6) | moderate |
| Akiful Haque | 0 | 1 | 0 | 1 | 0 | 1 | 1 | 1 | 0 | 5(55.6) | moderate |
| Alabdulla | 0 | 0 | 0 | 1 | 0 | 1 | 1 | 1 | 0 | 4(44.4) | high |
| AlAwadhi | 0 | 1 | 0 | 1 | 0 | 1 | 1 | 1 | 0 | 5(55.6) | moderate |
| Alfageeh | 0 | 1 | 0 | 1 | 0 | 1 | 1 | 1 | 1 | 6(66.7) | moderate |
| Alley | 0 | 0 | 0 | 1 | 0 | 1 | 1 | 1 | 0 | 4(44.4) | high |
| Al-Marshoudi | 0 | 0 | 1 | 1 | 1 | 1 | 1 | 1 | 1 | 7(77.8) | low |
| Al-Metwali | 0 | 0 | 0 | 1 | 0 | 1 | 1 | 1 | 0 | 4(44.4) | high |
| Al-Mistarehi | 0 | 0 | 1 | 1 | 1 | 1 | 1 | 1 | 1 | 7(77.8) | low |
| Al-Mohaithef | 0 | 1 | 1 | 1 | 1 | 1 | 1 | 1 | 1 | 8(88.9) | low |
| Al-Mulla | 0 | 0 | 1 | 1 | 1 | 1 | 1 | 1 | 1 | 7(77.8) | low |
| Alobaidi | 0 | 0 | 0 | 1 | 0 | 1 | 1 | 1 | 0 | 4(44.4) | high |
| Al-Qerem | 0 | 0 | 1 | 1 | 1 | 1 | 1 | 1 | 1 | 7(77.8) | low |
| Alqudeimat | 0 | 1 | 0 | 1 | 0 | 1 | 1 | 1 | 0 | 5(55.6) | moderate |
| Al-Sanafi | 0 | 1 | 1 | 1 | 1 | 1 | 1 | 1 | 1 | 8(88.9) | low |
| Alshahrani, | 0 | 1 | 1 | 1 | 1 | 1 | 1 | 1 | 1 | 8(88.9) | low |
| Alvarado-Socarras | 0 | 0 | 0 | 1 | 0 | 1 | 1 | 1 | 0 | 4(44.4) | high |
| Asadi Faezi | 0 | 0 | 0 | 1 | 0 | 1 | 1 | 1 | 0 | 4(44.4) | high |
| Attwell | 0 | 0 | 1 | 1 | 1 | 1 | 1 | 1 | 1 | 7(77.8) | low |
| Babicki | 0 | 0 | 0 | 1 | 0 | 1 | 1 | 1 | 1 | 5(55.6) | moderate |
| Baghdadi | 0 | 1 | 1 | 1 | 1 | 1 | 1 | 1 | 1 | 8(88.9) | low |
| Bai | 0 | 1 | 0 | 1 | 0 | 1 | 1 | 1 | 1 | 6(66.7) | moderate |
| Bendau | 0 | 1 | 0 | 1 | 0 | 1 | 1 | 1 | 0 | 5(55.6) | moderate |
| Blanchard-Rohner | 0 | 1 | 0 | 1 | 0 | 1 | 1 | 1 | 1 | 6(66.7) | moderate |
| Bongomin | 0 | 0 | 1 | 1 | 1 | 1 | 1 | 1 | 1 | 7(77.8) | low |
| Bono | 0 | 0 | 0 | 1 | 0 | 1 | 1 | 1 | 0 | 4(44.4) | high |
| Butter | 0 | 0 | 0 | 1 | 0 | 1 | 1 | 1 | 0 | 4(44.4) | high |
| Carmody | 0 | 1 | 0 | 1 | 0 | 1 | 1 | 1 | 0 | 5(55.6) | moderate |
| Cerda | 0 | 1 | 1 | 1 | 1 | 1 | 1 | 1 | 1 | 8(88.9) | low |
| Chaudhary, | 0 | 1 | 1 | 1 | 1 | 1 | 1 | 1 | 1 | 8(88.9) | low |
| Chen | 0 | 1 | 1 | 1 | 1 | 1 | 1 | 1 | 1 | 8(88.9) | low |
| Del Riccio | 0 | 1 | 0 | 1 | 0 | 1 | 1 | 1 | 0 | 5(55.6) | moderate |
| Detoc | 0 | 0 | 0 | 1 | 0 | 1 | 1 | 1 | 0 | 4(44.4) | high |
| Di Gennaro | 0 | 0 | 0 | 1 | 0 | 1 | 1 | 1 | 0 | 4(44.4) | high |
| Di Giuseppe | 1 | 1 | 1 | 1 | 1 | 1 | 1 | 1 | 1 | 9(100) | low |
| Dinga | 0 | 0 | 1 | 1 | 1 | 1 | 1 | 1 | 1 | 7(77.8) | low |
| Ditekemena | 0 | 0 | 0 | 1 | 0 | 1 | 1 | 1 | 0 | 4(44.4) | high |
| Dror | 0 | 0 | 0 | 1 | 0 | 1 | 1 | 1 | 0 | 4(44.4) | high |
| Dror | 0 | 0 | 0 | 1 | 0 | 1 | 1 | 1 | 0 | 4(44.4) | high |
| Dubé | 1 | 1 | 1 | 1 | 1 | 1 | 1 | 1 | 1 | 9(100) | low |
| Echoru | 0 | 1 | 1 | 1 | 1 | 1 | 1 | 1 | 1 | 8(88.9) | low |
| Edwards | 0 | 0 | 0 | 1 | 0 | 1 | 1 | 1 | 0 | 4(44.4) | high |
| Ehde | 0 | 1 | 0 | 1 | 0 | 1 | 1 | 1 | 0 | 5(55.6) | moderate |
| El-Elimat | 0 | 0 | 0 | 1 | 0 | 1 | 1 | 1 | 0 | 4(44.4) | high |
| Elhadi | 0 | 1 | 1 | 1 | 1 | 1 | 1 | 1 | 1 | 8(88.9) | low |
| Fares | 0 | 0 | 1 | 1 | 1 | 1 | 1 | 1 | 1 | 7(77.8) | low |
| Fedele | 0 | 0 | 0 | 1 | 0 | 1 | 1 | 1 | 0 | 4(44.4) | high |
| Fisher | 0 | 0 | 0 | 1 | 0 | 1 | 1 | 1 | 0 | 4(44.4) | high |
| Gagneux-Brunon | 0 | 0 | 0 | 1 | 0 | 1 | 1 | 1 | 0 | 4(44.4) | high |
| Gallè | 0 | 0 | 0 | 1 | 0 | 1 | 1 | 1 | 0 | 4(44.4) | high |
| Gan | 0 | 1 | 1 | 1 | 1 | 1 | 1 | 1 | 1 | 8(88.9) | low |
| Gatwood | 0 | 0 | 1 | 1 | 1 | 1 | 1 | 1 | 1 | 7(77.8) | low |
| Graﬃgna | 0 | 0 | 0 | 1 | 0 | 1 | 1 | 1 | 0 | 4(44.4) | high |
| Grochowska | 0 | 0 | 0 | 1 | 0 | 1 | 1 | 1 | 0 | 4(44.4) | high |
| Guaraldi | 0 | 0 | 0 | 1 | 0 | 1 | 1 | 1 | 0 | 4(44.4) | high |
| Hammer | 1 | 1 | 1 | 1 | 1 | 1 | 1 | 1 | 1 | 9(100) | low |
| Han | 1 | 0 | 0 | 1 | 0 | 1 | 1 | 1 | 0 | 5(55.6) | moderate |
| Handebo | 1 | 1 | 1 | 1 | 1 | 1 | 1 | 1 | 1 | 9(100) | low |
| Harapan | 1 | 1 | 1 | 1 | 1 | 1 | 1 | 1 | 1 | 9(100) | low |
| Hetherington | 0 | 0 | 1 | 1 | 1 | 1 | 1 | 1 | 1 | 7(77.8) | low |
| Holeva | 0 | 1 | 0 | 1 | 0 | 1 | 1 | 1 | 0 | 5(55.6) | moderate |
| Huynh | 0 | 1 | 1 | 1 | 1 | 1 | 1 | 1 | 1 | 8(88.9) | low |
| İkiışık | 1 | 1 | 1 | 1 | 1 | 1 | 1 | 1 | 0 | 8(88.9) | low |
| Jacob | 0 | 1 | 0 | 1 | 0 | 1 | 1 | 1 | 0 | 5(55.6) | moderate |
| Janssen | 0 | 0 | 0 | 1 | 0 | 1 | 1 | 1 | 0 | 4(44.4) | high |
| Jaramillo-Monge | 0 | 0 | 0 | 1 | 0 | 1 | 1 | 1 | 0 | 4(44.4) | high |
| Johnson | 0 | 0 | 0 | 1 | 0 | 1 | 1 | 1 | 0 | 4(44.4) | high |
| Kabamba | 0 | 0 | 0 | 1 | 0 | 1 | 1 | 1 | 0 | 4(44.4) | high |
| Kadoya | 0 | 0 | 0 | 1 | 0 | 1 | 1 | 1 | 0 | 4(44.4) | high |
| Kanyike | 0 | 1 | 0 | 1 | 0 | 1 | 1 | 1 | 0 | 5(55.6) | moderate |
| Kaplan | 0 | 1 | 1 | 1 | 1 | 1 | 1 | 1 | 1 | 8(88.9) | low |
| Kasrine | 0 | 1 | 1 | 1 | 1 | 1 | 1 | 1 | 1 | 8(88.9) | low |
| Kelkar | 0 | 0 | 0 | 1 | 0 | 1 | 1 | 1 | 0 | 4(44.4) | high |
| Khaled | 1 | 1 | 0 | 1 | 0 | 1 | 1 | 1 | 0 | 6(66.7) | moderate |
| Khubchandani | 0 | 0 | 1 | 1 | 1 | 1 | 1 | 1 | 1 | 7(77.8) | low |
| Konopi´nska | 0 | 0 | 1 | 1 | 1 | 1 | 1 | 1 | 1 | 7(77.8) | low |
| Kourlaba | 1 | 1 | 1 | 1 | 1 | 1 | 1 | 1 | 1 | 9(100) | low |
| Kumari | 0 | 1 | 0 | 1 | 0 | 1 | 1 | 1 | 0 | 5(55.6) | moderate |
| Kuter | 0 | 1 | 0 | 1 | 0 | 1 | 1 | 1 | 0 | 5(55.6) | moderate |
| Kwok | 0 | 0 | 1 | 1 | 1 | 1 | 1 | 1 | 1 | 7(77.8) | low |
| La Vecchia | 0 | 0 | 0 | 0 | 0 | 1 | 1 | 1 | 0 | 3(33.3) | high |
| Lamptey | 0 | 1 | 1 | 1 | 1 | 1 | 1 | 1 | 1 | 8(88.9) | low |
| Ledda | 0 | 0 | 0 | 1 | 0 | 1 | 1 | 1 | 0 | 4(44.4) | high |
| Lin | 0 | 0 | 0 | 1 | 0 | 1 | 1 | 1 | 0 | 4(44.4) | high |
| Liu D | 0 | 0 | 0 | 1 | 0 | 1 | 1 | 1 | 0 | 4(44.4) | high |
| Liu T | 0 | 1 | 0 | 1 | 0 | 1 | 1 | 1 | 0 | 5(55.6) | moderate |
| Lucia | 0 | 0 | 0 | 1 | 0 | 1 | 1 | 1 | 0 | 4(44.4) | high |
| Luk | 1 | 1 | 0 | 1 | 0 | 1 | 1 | 1 | 0 | 6(66.7) | moderate |
| Machida | 0 | 1 | 0 | 1 | 0 | 1 | 1 | 1 | 0 | 5(55.6) | moderate |
| Malesza | 0 | 1 | 0 | 1 | 0 | 1 | 1 | 1 | 0 | 5(55.6) | moderate |
| Malik | 0 | 0 | 1 | 1 | 1 | 1 | 1 | 1 | 1 | 7(77.8) | low |
| Maraqa | 0 | 1 | 1 | 1 | 1 | 1 | 1 | 1 | 1 | 8(88.9) | low |
| Mascarenhas | 0 | 0 | 0 | 1 | 0 | 1 | 1 | 1 | 0 | 4(44.4) | high |
| Mesele | 1 | 1 | 1 | 1 | 1 | 1 | 1 | 1 | 1 | 9(100) | low |
| Mohamad | 0 | 0 | 0 | 1 | 0 | 1 | 1 | 1 | 0 | 4(44.4) | high |
| Mose | 1 | 1 | 1 | 1 | 1 | 1 | 1 | 1 | 1 | 9(100) | low |
| Murphy | 0 | 0 | 1 | 1 | 1 | 1 | 1 | 1 | 1 | 7(77.8) | low |
| Nikolovski | 0 | 0 | 0 | 1 | 0 | 1 | 1 | 1 | 0 | 4(44.4) | high |
| Nohl | 0 | 0 | 0 | 1 | 0 | 1 | 1 | 1 | 0 | 4(44.4) | high |
| Olanipekun | 0 | 0 | 0 | 1 | 0 | 1 | 1 | 1 | 0 | 4(44.4) | high |
| Palamenghi | 0 | 1 | 0 | 1 | 0 | 1 | 1 | 1 | 0 | 5(55.6) | moderate |
| Panda | 0 | 1 | 1 | 1 | 1 | 1 | 1 | 1 | 1 | 8(88.9) | low |
| Parente | 0 | 0 | 0 | 1 | 0 | 1 | 1 | 1 | 0 | 4(44.4) | high |
| Pataka | 0 | 0 | 0 | 1 | 0 | 1 | 1 | 1 | 0 | 4(44.4) | high |
| Piltch-Loeb | 0 | 1 | 0 | 1 | 0 | 1 | 1 | 1 | 0 | 5(55.6) | moderate |
| Pogue | 0 | 0 | 0 | 1 | 0 | 1 | 1 | 1 | 0 | 4(44.4) | high |
| Prati | 0 | 1 | 0 | 1 | 0 | 1 | 1 | 1 | 0 | 5(55.6) | moderate |
| Puteikis | 0 | 0 | 0 | 1 | 0 | 1 | 1 | 1 | 0 | 4(44.4) | high |
| Qattan | 0 | 1 | 0 | 1 | 0 | 1 | 1 | 1 | 0 | 5(55.6) | moderate |
| Qin | 1 | 1 | 0 | 1 | 0 | 1 | 1 | 1 | 0 | 6(66.7) | moderate |
| Rabi | 0 | 0 | 1 | 1 | 1 | 1 | 1 | 1 | 1 | 7(77.8) | low |
| Racey | 0 | 0 | 1 | 1 | 1 | 1 | 1 | 1 | 1 | 7(77.8) | low |
| Reiter | 0 | 1 | 0 | 1 | 0 | 1 | 1 | 1 | 0 | 5(55.6) | moderate |
| Reno | 0 | 1 | 0 | 1 | 0 | 1 | 1 | 1 | 0 | 5(55.6) | moderate |
| Riad | 0 | 0 | 1 | 1 | 1 | 1 | 1 | 1 | 1 | 7(77.8) | low |
| Rodríguez-Blanco | 0 | 0 | 1 | 1 | 1 | 1 | 1 | 1 | 1 | 7(77.8) | low |
| Saied | 0 | 1 | 1 | 1 | 1 | 1 | 1 | 1 | 1 | 8(88.9) | low |
| Sallam | 0 | 0 | 0 | 1 | 0 | 1 | 1 | 1 | 0 | 4(44.4) | high |
| Sallam_b | 0 | 1 | 1 | 1 | 1 | 1 | 1 | 1 | 1 | 8(88.9) | low |
| Salmon | 1 | 1 | 0 | 1 | 0 | 1 | 1 | 1 | 0 | 6(66.7) | moderate |
| Schwarzinger | 0 | 1 | 0 | 1 | 0 | 1 | 1 | 1 | 0 | 5(55.6) | moderate |
| **Seale** | 0 | 1 | 1 | 1 | 1 | 1 | 1 | 1 | 1 | 8(88.9) | low |
| Serrazina | 0 | 0 | 0 | 1 | 0 | 1 | 1 | 1 | 0 | 4(44.4) | high |
| Sharma | 0 | 0 | 1 | 1 | 1 | 1 | 1 | 1 | 1 | 7(77.8) | low |
| Shaw | 0 | 0 | 0 | 1 | 0 | 1 | 1 | 1 | 0 | 4(44.4) | high |
| Shekhar | 0 | 1 | 0 | 1 | 0 | 1 | 1 | 1 | 0 | 5(55.6) | moderate |
| Sherman | 0 | 1 | 1 | 1 | 1 | 1 | 1 | 1 | 1 | 8(88.9) | low |
| Shih | 1 | 1 | 1 | 1 | 1 | 1 | 1 | 1 | 1 | 9(100) | low |
| Shmueli | 0 | 0 | 0 | 1 | 0 | 1 | 1 | 1 | 0 | 4(44.4) | high |
| Skjefte | 0 | 0 | 0 | 1 | 0 | 1 | 1 | 1 | 0 | 4(44.4) | high |
| Soares | 0 | 0 | 0 | 1 | 0 | 1 | 1 | 1 | 0 | 4(44.4) | high |
| Stern | 0 | 0 | 0 | 1 | 0 | 1 | 1 | 1 | 0 | 4(44.4) | high |
| Stuckelberger | 0 | 0 | 0 | 1 | 0 | 1 | 1 | 1 | 0 | 4(44.4) | high |
| Sun | 0 | 0 | 0 | 1 | 0 | 1 | 1 | 1 | 0 | 4(44.4) | high |
| Syed Alwi | 0 | 1 | 1 | 1 | 1 | 1 | 1 | 1 | 1 | 8(88.9) | low |
| Talarek | 0 | 0 | 0 | 1 | 0 | 1 | 1 | 1 | 0 | 4(44.4) | high |
| Tao | 0 | 1 | 1 | 1 | 1 | 1 | 1 | 1 | 1 | 8(88.9) | low |
| Tavolacci | 0 | 1 | 0 | 1 | 0 | 1 | 1 | 1 | 0 | 5(55.6) | moderate |
| Temsah | 0 | 1 | 0 | 1 | 0 | 1 | 1 | 1 | 0 | 5(55.6) | moderate |
| Thaker | 0 | 1 | 0 | 1 | 0 | 1 | 1 | 1 | 0 | 5(55.6) | moderate |
| Trabucco Aurilio | 0 | 0 | 0 | 1 | 0 | 1 | 1 | 1 | 0 | 4(44.4) | high |
| Tran | 0 | 1 | 1 | 1 | 1 | 1 | 1 | 1 | 1 | 8(88.9) | low |
| Tsai | 0 | 1 | 0 | 1 | 0 | 1 | 1 | 1 | 0 | 5(55.6) | moderate |
| Tsapepas | 0 | 0 | 0 | 1 | 0 | 1 | 1 | 1 | 0 | 4(44.4) | high |
| Urrunaga-Pastor | 0 | 0 | 0 | 1 | 0 | 1 | 1 | 1 | 0 | 4(44.4) | high |
| Vallée | 0 | 0 | 0 | 1 | 0 | 1 | 1 | 1 | 0 | 4(44.4) | high |
| Viswanath | 1 | 1 | 1 | 1 | 1 | 1 | 1 | 1 | 1 | 9(100) | low |
| Walker | 0 | 1 | 0 | 1 | 0 | 1 | 1 | 1 | 0 | 5(55.6) | moderate |
| Wang C | 0 | 0 | 0 | 1 | 0 | 1 | 1 | 1 | 0 | 4(44.4) | high |
| Wang J_a | 0 | 1 | 0 | 1 | 0 | 1 | 1 | 1 | 0 | 5(55.6) | moderate |
| Wang J_b | 1 | 1 | 0 | 1 | 0 | 1 | 1 | 1 | 0 | 6(66.7) | moderate |
| Wang K_a | 0 | 0 | 0 | 1 | 0 | 1 | 1 | 1 | 0 | 4(44.4) | high |
| Wang K_b | 0 | 0 | 0 | 1 | 0 | 1 | 1 | 1 | 0 | 4(44.4) | high |
| Williams | 0 | 1 | 0 | 1 | 0 | 1 | 1 | 1 | 0 | 5(55.6) | low |
| Wirawan | 0 | 0 | 0 | 1 | 0 | 1 | 1 | 1 | 0 | 4(44.4) | high |
| Wong L | 0 | 0 | 0 | 1 | 0 | 1 | 1 | 1 | 0 | 4(44.4) | high |
| Wong M | 1 | 1 | 0 | 1 | 0 | 1 | 1 | 1 | 0 | 6(66.7) | moderate |
| Xiang | 0 | 0 | 0 | 1 | 0 | 1 | 1 | 1 | 0 | 4(44.4) | high |
| Xu | 0 | 0 | 0 | 1 | 0 | 1 | 1 | 1 | 0 | 4(44.4) | high |
| Yan | 1 | 1 | 0 | 1 | 0 | 1 | 1 | 1 | 0 | 6(66.7) | moderate |
| Yang F | 0 | 1 | 1 | 1 | 1 | 1 | 1 | 1 | 1 | 8(88.9) | low |
| Yang Y | 0 | 0 | 0 | 1 | 0 | 1 | 1 | 1 | 0 | 4(44.4) | high |
| Yılmaz | 0 | 0 | 1 | 1 | 1 | 1 | 1 | 1 | 1 | 7(77.8) | low |
| Yurttas | 0 | 1 | 0 | 1 | 0 | 1 | 1 | 1 | 0 | 5(55.6) | moderate |
| Zewude | 0 | 1 | 1 | 1 | 1 | 1 | 1 | 1 | 1 | 8(88.9) | low |
| Zhang | 0 | 0 | 1 | 1 | 1 | 1 | 1 | 1 | 0 | 6(66.7) | moderate |
